# Minor Sea Turtle Nesting Areas May Remain Unnoticed without Specific Monitoring: The Case of the Largest Mediterranean Island (Sicily, Italy)

**DOI:** 10.3390/ani12091221

**Published:** 2022-05-09

**Authors:** Oleana Olga Prato, Valentina Paduano, Giulia Baldi, Salvatore Bonsignore, Gerlando Callea, Carlo Camera, Girolamo Culmone, Stefania D’angelo, Diego Fiorentino, Gino Galia, Salvatore Coriglione, Laura Genco, Giuseppe Mazzotta, Nicola Napolitano, Francesco Paolo Palazzo, Giuseppe Palilla, Santo Dylan Pelletti, Toni Mingozzi, Luigi Agresti, Paolo Casale

**Affiliations:** 1 WWF Italy, Via Po, 25c, 00198 Roma, Italy; oleanaolga@gmail.com (O.O.P.); valentina.paduano22@gmail.com (V.P.); s.bonsignore@wwf.it (S.B.); g.callea@wwf.it (G.C.); carlocamera@hotmail.com (C.C.); s.dangelo@wwf.it (S.D.); diego.fiorentino72@gmail.com (D.F.); galiagino@libero.it (G.G.); dott.salvatorecoriglione@gmail.com (S.C.); l.genco@wwf.it (L.G.); g.mazzotta54@gmail.com (G.M.); n.napolitano@wwf.it (N.N.); fp_palazzo@msn.com (F.P.P.); g.palilla@wwf.it (G.P.); dilanpelletti@icloud.com (S.D.P.); l.agresti@wwf.it (L.A.); 2 Department of Biology, University of Pisa, Via A. Volta 6, 56126 Pisa, Italy; bald.giu@gmail.com; 3 Associazione Caretta Caretta, Via L. Ariosto 86, 92031 Lampedusa, Italy; geogiro@libero.it; 4 DiBEST, Department of Biology, Ecology and Earth Sciences, Università della Calabria, P.te P. Bucci, Cubo 4/B, 87030 Rende, Italy; antonio.mingozzi@unical.it

**Keywords:** *Caretta caretta*, loggerhead turtle, incubation period, clutch size, hatching success, emergence success, conservation

## Abstract

**Simple Summary:**

Before protecting sea turtles’ nesting sites from coastal development, these sites must be identified and evaluated. This is particularly difficult with minor nesting sites distributed over large areas. We report on the case of Sicily, the largest Mediterranean island with 464 km of sandy shores, where sea turtle nesting activity was basically unknown until recent years when specific projects focused on this topic. This may be the case for many other areas. A total of 323 nests have been reported in the 1944–2021 period (mostly in the last decade). However, the real number of nests occurring annually is still unknown and more research and monitoring is needed. In sea turtles, sex is determined by the incubation temperature, with high temperatures producing more females, and with global warming the scarcity of males may become a problem. Nests in Sicily seem to produce more males and therefore this area may be important for the species’ conservation in the future.

**Abstract:**

Identifying coastal tracts suitable for sea turtle reproduction is crucial for sea turtle conservation in a context of fast coastal development and climate change. In contrast to nesting aggregations, diffuse nesting is elusive and assessing nesting levels is challenging. A total of 323 nesting events by the loggerhead sea turtle *Caretta caretta* have been reported in Sicily, the largest Mediterranean island, in the 1944–2021 period, mostly in the last decade. Specific monitoring efforts are the most likely explanation for such an increase and shows that sea turtle nesting may be underestimated or completely ignored in many areas with scattered nesting. The real nesting level along the 464 km sandy shores of Sicily is still unknown and more research is needed. The observed incubation period was relatively long (57 d) suggesting that a majority of males are produced in Sicily, in contrast to the typical female-biased sex ratio of sea turtles. In a context of climate warming producing sex ratios more skewed towards females, the potential of Sicily as a male-producing area should be further investigated. Other reproductive parameters are provided, such as clutch size and hatching and emergence success. A negative effect of relocation on the latter two was observed.

## 1. Introduction

Sea turtle populations range across wide foraging areas at sea but breed in fewer and much more localized areas, where females lay egg clutches on sandy beaches [1]. Such aggregations of a critical life stage represent both a vulnerability and an opportunity for conservation of these threatened animals. Degradation of nesting habitat and disturbance of nesting females, of egg incubation and of hatchlings while going to the sea are considered as important anthropogenic threats worldwide e.g., [2].

In the Mediterranean Sea, the most common sea turtle species, the loggerhead sea turtle *Caretta caretta*, typically breeds in a few sites with high clutch densities. These “major” nesting sites are localized in Greece, Turkey, Cyprus, and Libya [3]. However, “minor” nesting sites occur in several other countries and individual clutches can be found almost all over the basin [3]. With an increasing anthropogenic pressure along the coasts of the Mediterranean basin [4], identifying coastal tracts suitable for sea turtle nesting before they are developed in an irreversible way is crucial for sea turtle conservation. This is particularly relevant in the context of climate change [5] that can induce different distribution of nesting activity in the future [6].

In spite of decades of conservation interest in these animals, the current knowledge about the distribution of sea turtle nesting activity is still far to be complete, and even major nesting sites may be discovered [7]. Assessing actual nesting levels is much more difficult with relatively low clutch density. For instance, while it is unlikely that Italy hosts any major nesting sites of *Caretta caretta*, the existence of the Calabrian Ionian nesting area was unknown until a specific project was carried out [8]. An analogous case was suspected in Sicily when the number of reported clutches suddenly increased after a citizen science campaign [9]. Sicily is the largest island of the Mediterranean Sea, surrounded by the Tyrrhenian Sea, the Ionian Sea, and the Sicily Channel. With over 1100 km of coastline, it bears great potential regarding sea turtle nesting levels, although just a few nests were reported in the past [8].

In the last decade, more efforts have been allocated on detecting sea turtle clutches laid along the coasts of Sicily. Beach surveys have been intensified and expanded to new areas, and local people have become more informed about sea turtle nesting and how to report a track. Such a positive trend was further boosted by a specific 5-years project (2017–2021; LIFE Euroturtles; www.euroturtles.eu (accessed on 1 March 2022) that increased technical capacity and monitoring efforts.

This study aims to provide a more realistic assessment of the nesting level in the island of Sicily and its conservation implications in the Mediterranean context, through a comprehensive analysis of all of the sea turtle nesting activity reported so far, including the results of the recent monitoring project.

## 2. Materials and Methods

### 2.1. Data Collection

Sea turtle nesting events were mainly recorded opportunistically, through reports by tourists, local people, local authorities or NGOs. Such records were provided to this study either directly by the persons or organizations that collected the scientific data or indirectly through a literature review, online articles and interviews. The complete list of records and sources is provided in Supplemental Table S1. In a few areas and periods periodical on foot surveys specifically aimed to detect sea turtle nests were carried out. A more intensive monitoring was carried out by the authors in the 2017–2020 period in the framework of a specific project (LIFE Euroturtles). However, even in this case the extent of the coast prevented a uniform monitoring and some nests were found only after hatching. The following description regards this specific project (LIFE Euroturtles, 2017–2020). When possible, from June to August beaches were monitored during the first hours of light (generally from 5 to 8 a.m.) to detect tracks of sea turtles emerging from the sea. Part of the events were reported by passerby or beach operators.

From external examination (and later confirmation of presence of eggs), each track was assigned to two categories: “nest” (with eggs) or “false crawl”. The distance of the nest from the shoreline (DIST) was measured. If a nest was at risk of being washed over, it was relocated further from the sea. A few nests were relocated for avoiding their destruction by human activities. Most nests were relocated the same day of nesting, while a few nests were relocated when they were inundated or damaged. In the most frequented beaches, the nest was protected by a gridded fence. For nests that were found at night upon deposition, date of nesting was assigned as the date of the following morning. Hatchling emergence was detected by monitoring the nest. In some cases, unknown nests were discovered through hatchling emergences reported by passerby. The nest was excavated after minimum three days after the last emergence. In case of eggs still developing, they were left in the chamber. The number of hatched eggs (from fragments larger than 50% of an eggshell; [10]), unhatched eggs, live and dead hatchlings in the chamber were recorded.

### 2.2. Data Analysis

Incubation Period (IP, in days) was calculated as the number of days between nesting and first emergence date for the nests where this information was available. Hatching success (*HS*) and emergence success (*ES*) were calculated as:

(1) $$HS=\frac{H}{CS}\;$$

(2) $$ES=\frac{\left(H-NE\right)}{CS}\;$$

where *H* is the number of hatched eggs from fragments of shells, *NE* (not emerged) is the number of live and dead hatchlings remained in the nest and *CS* is the clutch size (total number of eggs laid).

**Figure 1:**
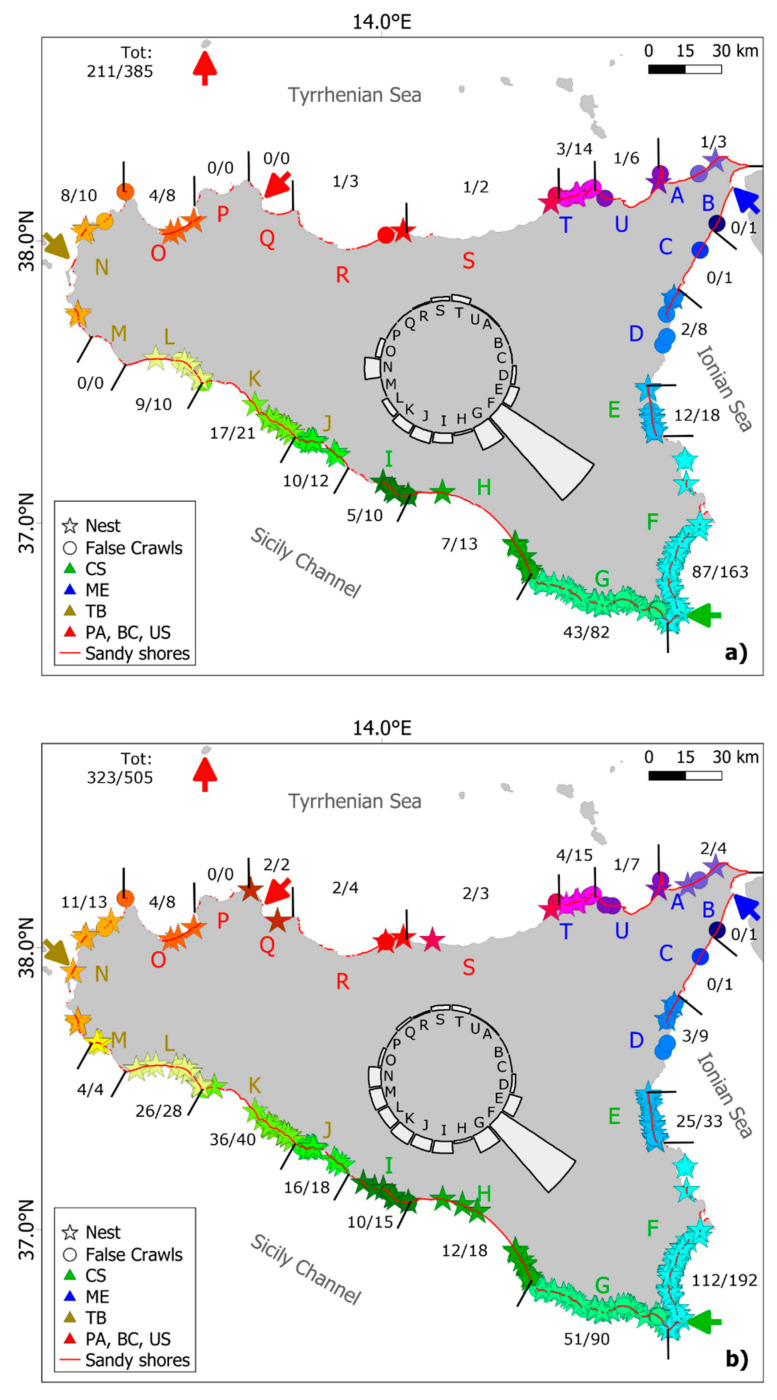

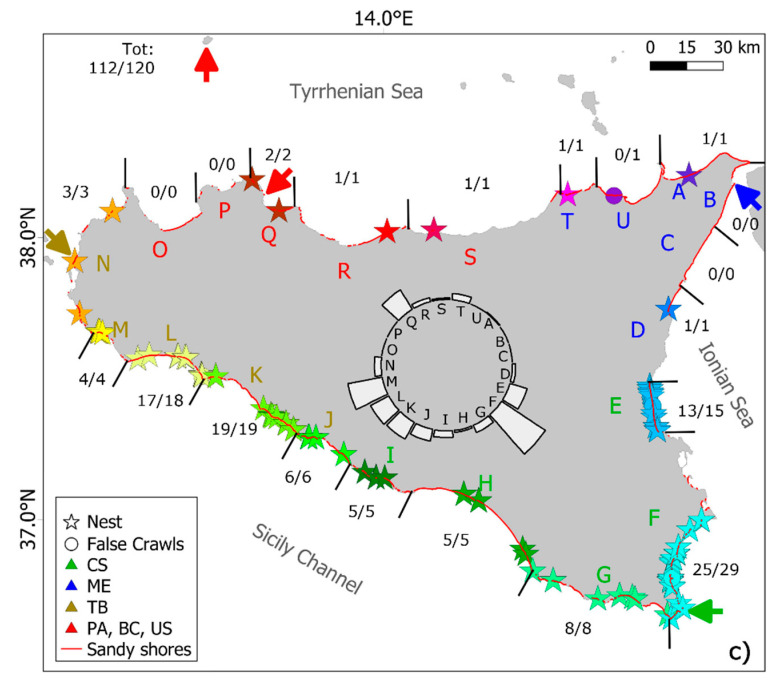
Sea turtle emergences reported in Sicily from 2017 to 2021 (**a**), 1944 to 2021 (**b**) and 1944 to 2016 (**c**), divided in Nests (stars) and False crawls (circles). Black lines perpendicular to the perimeter of the island indicate the 21 Physiographic Units (PU) identified by letters. The number of nests and the total number of emergences (nests + false crawls) are provided for each PU (in the form nests/emergences) and the totals are given in the top left of the figure. PUs letters are color coded with the Temperature Stations (arrows; Trapani Birgi: TB, Palermo: PA, Ustica Island: US, Boccadifalco: BC, Messina: ME, Capo Rizzuto: CR) that originated the data that were used to calculate IPMT. The circular plot in the middle provides mean annual nest density (nests km-1 year-1) for each PU.

For convenience, we divided the coast according to the 21 Physiographic Units (PU) determined by Regione Sicilia (2004; https://www.sitr.regione.sicilia.it/pai/index.htm (accessed on 22 October 2021); Figure 1) based on geomorphological characteristics. In QGIS [11], coast lines data (ISTAT, 2013; https://www.istat.it/it/archivio/137341 (accessed on 12 November 2021)) consisting of segments delimiting the land perimeter of the island were visually inspected to designate sandy coast. For each PU, the cumulative length of sandy coast (including sand and sand mixed with pebbles considered suitable for nesting) was estimated by the sum of the selected segments. This length of sandy shores was used to calculate the observed nesting frequency (nests km-1 yr-1) per PU.

Sand of different colors has different capacity to absorb or refract light: at the same environmental conditions, a darker sand absorbs more light and has a higher temperature [12]. In the absence of nests temperature data, we assigned sand colours (SC) for each beach as a proxy. A form was prepared with one picture at maximum zoom from Google Earth and two picture of the locality of a sample of several beaches per PU. Five color classes (WH, white; YL, yellow; GR, gray; OC, ochre; BL, black) were chosen from a previous general assessment of the beaches in Sicily. The form was distributed among collaborators to objectively assign specific color characteristics to each class. Ultimately, a color class was assigned to each location where nests occurred.

Air temperature data of the period 2000–2020 were obtained from NOAA (https://www.ncdc.noaa.gov/cdo-web/ (accessed on 26 October 2021)), with six stations available in the study area: Trapani Birgi (TB), Palermo (PA), Ustica Island (US), Boccadifalco (BC), Messina (ME), Capo Rizzuto (CR). PUs were assigned to each station according to their distance (stations PA, US and BC were grouped due to scarcity of data). The dataset presented daily minimum and maximum temperature, when both were available the average was also listed. Incubation Period mean temperature (IPMT) was calculated for nesting events for which nesting and hatching date were known, averaging daily mean temperatures between the two dates.

To understand how IP and *HS* of natural nests are influenced by environmental parameters, a Generalized Linear Model (GLM, glm function of stats package) with a gamma distribution was used for IP, while a binomial GLM with cloglog link was used for *HS* (to account for the high number of zeros) [13]. In both cases, the following formula was used: Dependent Variable ~ PU + YEAR + DATE * IPMT + SC + DIST + CS (where DATE is the date of nesting). For categorical variables, the category with the largest sample size was considered as the first (intercept) level. Then, a stepwise algorithm was used to reduce model complexity (step function of stats package, direction: backward) and the model with the lowest Akaike information criterion (AIC) was selected. All analyses were performed in R [14].

To provide insights about the potential primary sex ratio produced by the observed nests, IP was used as a proxy of incubation temperature. The value of 56 days, obtained from loggerhead turtles in Cyprus [15], was considered as the Pivotal Incubation Period (PIP; the incubation period associated to the production of a balanced sex ratio).

To investigate the effect of relocation, natural and relocated nests were compared through a Fisher exact test conducted on *HS* (hatched eggs vs. non hatched) and ES (emerged hatchlings vs. non emerged), and through a Mann-Whitney test conducted on CS, IP, IPMT. Given that a few nests were relocated after inundation or damage, only nests relocated before any impact were included in this analysis. In natural nests, a potential relationship between CS and DIST was tested with a Pearson chi-squared test.

## 3. Results

### 3.1. Spatio-Temporal Distribution of Sea Turtle Nesting Activity

**Figure 2:**
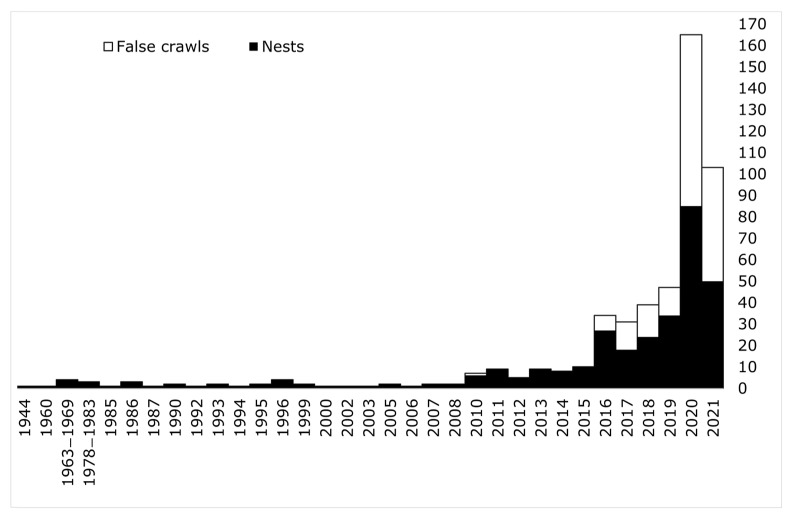
Temporal distribution of loggerhead turtle nests (*n* = 323) and false crawls (*n* = 180) recorded in Sicily (Italy) in the 1944–2021 period. Grouped years are reported as in the original source of the data [8].

**Figure 3:**
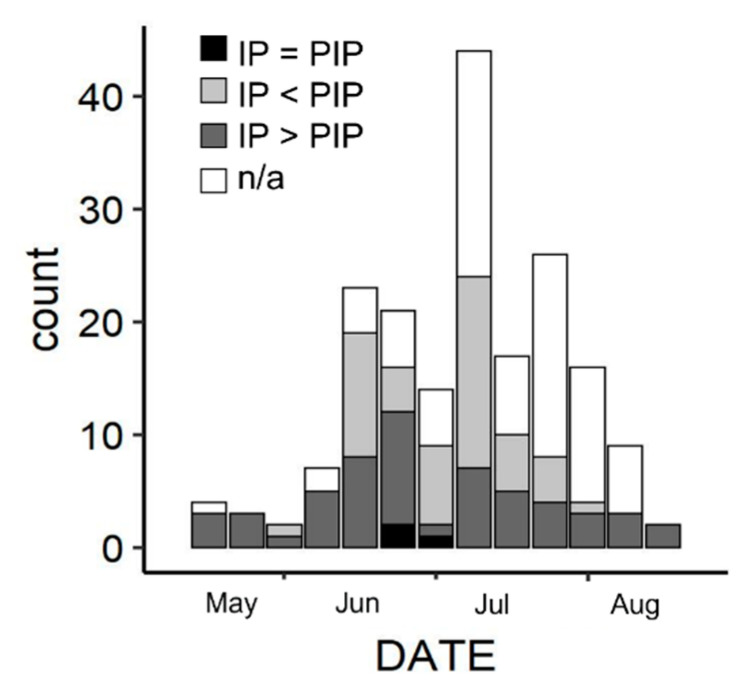
Temporal distribution of loggerhead turtle nesting date (DATE) in Sicily (*n* = 188). IP: incubation period; PIP, IP potentially producing and equal number of males and females; n/a, IP not available.

A total of 505 sea turtle emergences, of which 323 nests, have been reported in Sicily from 1944 to 2021 (Figure 1 and Figure 2). A total of 112 nests (34.7%) occurred in coastal tracts included in Natura 2000 sites (Supplemental Table S1). All hatchlings and eggs directly observed by experts belonged to the loggerhead turtle and no track resembled that of other species. Therefore, we assume that all of the present records regard loggerhead turtles only. Most nests (55.8%) were recorded in PUs F and G only. No nesting was reported from PUs B, C, P, although one false crawl was recorded in PUs B and C. Nesting date ranged between 25 May and 18 August, with a peak in mid-July (Figure 3). First hatchling emergence ranged between 26 July and 4 November (median: 3 September). Up to 18 days elapsed between the first and the last hatchling emergence from the same nest.

Before 2017, five nests were relocated and only eight false crawls were reported, while in the monitored period (2017–2021), 37 nests were relocated and more attention was given to false crawls (174 reported). In the 2017–2021 period, 55 nests were found through monitoring, while 103 nests were reported by beach goers and beach workers.

Sandy shores amounted to 464.3 km in length, representing from 6.6 to 81.4% of the PU coasts. Nests were found mainly in Ochre (OC; distributed almost entirely from PU E to L) or yellow beaches (YL; predominant in PUs E and F but also scattered throughout the coasts). Gray (GR) nesting beaches were limited (mostly north and northeast coast, PUs R to D). White (WH) nesting beaches were few and restricted to some locations in PU A and between PUs M and R. No nests were reported in the few black (BL) beaches located in the Ionian coast (Table 1).

Mean annual nest density for the different PUs was 0.10 nests km-1 yr-1 (range: 0–1.02) and 0.01 nests km-1 yr-1 (range: 0–0.08) for the period 2017–2021 and the entire period, respectively. Temporal continuity of nesting was spatially limited to PU from D to L where nesting occurred regularly (at least one nest every other year between 2017–2021).

Fifty-five nests had IP > PIP (potentially producing more males), 50 nests had IP < PIP (potentially producing more females), and three nest had IP = PIP (potentially producing an equal number of males and females). Their seasonal and geographic distribution are shown in Figure 3 and Figure S1, respectively.

### 3.2. Reproductive Parameters

**Figure 4:**
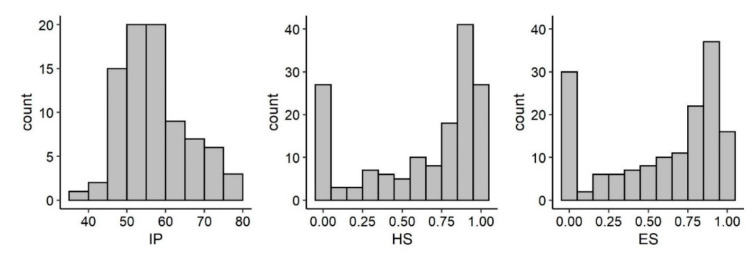
Frequency distribution of IP (*n* = 83), *HS* (*n* = 155), ES (*n* = 155) in loggerhead turtle natural nests in Sicily.

**Figure 5:**
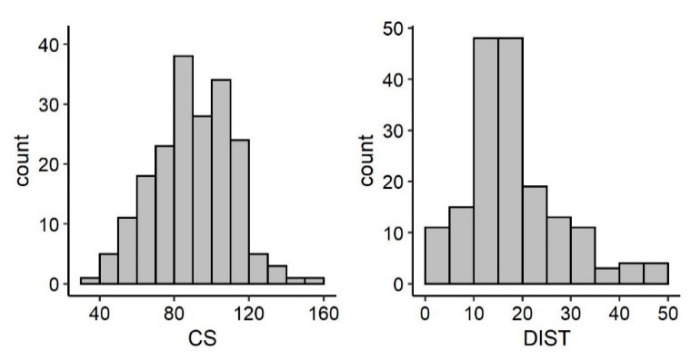
Frequency distribution of CS (*n* = 192) and DIST (*n* = 176) for all loggerhead turtle nests in Sicily.

**Figure 6:**
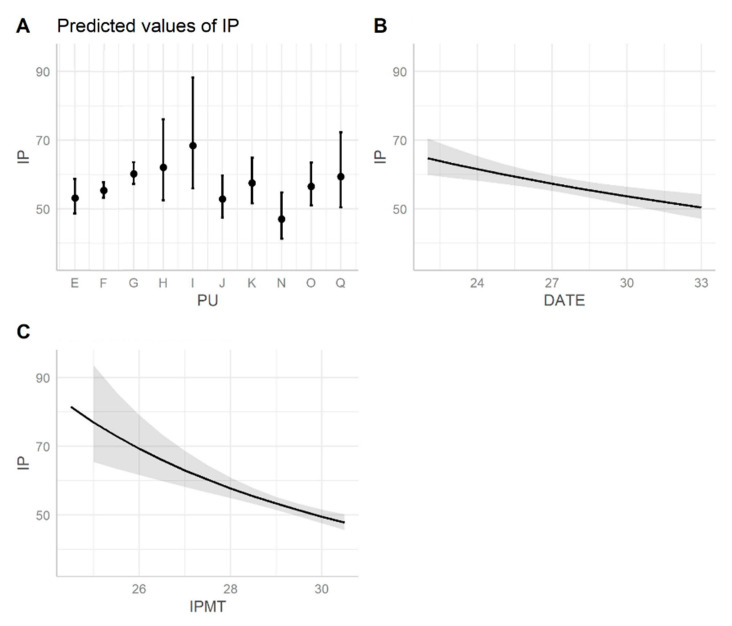
Predicted values of IP (estimated marginal means) of loggerhead turtle nests in Sicily plotted separately to show the effect of each IV, originated from the fittest GLM model. Dots in plot (**A**) show the predicted value for each PU, whiskers represent 95% CI. Plot (**B**,**C**) represent regression lines based on the GLM model with 95% CI in light gray. DATE: date of nesting (week of the year). IPMT: mean air temperature during the incubation period (°C).

Reproductive parameters of natural and relocated nests are shown in Table 1, Figure 3, Figure 4 and Figure 5. The fittest models for IP and *HS* resulting from the stepwise regression were, respectively, IP ~ PU + DATE + IPMT + CS + DATE:IPMT and *HS* ~ PU + YEAR + DATE * IPMT + SC + DIST + CS. These are the models including factors affecting IP and *HS* and the significant main effects are summarized in Supplemental Tables S2 and S3, respectively. The effect of each IV on the DV is shown in Figure 6 and Figure S3, respectively.

The nests relocated before any damage (*n* = 23) were relocated the same day of nesting, except fot 5 and 1 nests that were relocated after 1 or 2 days, respectively. Relocated nests had lower *HS* and ES (Fisher’s Exact Test, *HS*: *p* < 0.001, 95% CI = 1.51–1.83, odds ratio = 1.67, *n* = 23 relocated, 155 natural; ES: *p* < 0.01, 95% CI = 1.07–1.66, odds ratio =1.34, *n* = 23 relocated, 155 natural) than natural nests, while no significant difference was observed between the two groups on CS (Mann-Whitney U = 1999.5, *p* = 0.35, *n* = 23 relocated, 155 natural), IP (Mann-Whitney U = 751, *p* = 75, *n* = 19 relocated, 83 natural) and on IPMT (Mann-Whitney U = 797, *p* = 0.51, *n* = 20 relocated, 88 natural). CS and DIST in natural nests were significantly correlated (R = 0.26, *t* = 3.07, *p*-value = 0.003, *n* = 127; Figure S2).

## 4. Discussion

This study shows how the monitoring level can dramatically change the perception of an area in terms of sea turtle nesting activity and conservation importance. Sea turtle nesting may be underestimated or completely ignored in many areas with scattered nesting if dedicated surveys are not implemented. It is now evident that the island of Sicily hosts regular sea turtle nesting sites, as previously hypothesized [8].

The observed increase of nest numbers in the last ten years could be caused either by a real increase of nests in Sicily, by a higher monitoring effort or by a combination of the two. Although positive trends of nest counts have been observed in several Mediterranean nesting sites, they were not so fast and intense [3]. The observed tenfold increase in Sicily in such a short period cannot reflect a similar population increase in species with long generation length such as sea turtles. An alternative explanation for a real nest increase could be a shift of nesting activity from other areas, induced by unknown factors, possibly including climate change [16]. However, there are no major sea turtle nesting sites near Sicily, and this scenario would require females to shift from very distant Mediterranean nesting sites (e.g., Libya or Greece) [3] or even from the Atlantic, given that some of the clutches recently found in the western Mediterranean were laid by Atlantic females [17]. However, the nesting activity observed in the western Mediterranean is low and any relation to the increase observed in Sicily is uncertain. Unfortunately, current genetic data about nests in Sicily are not much informative [9], with an mtDNA haplotype shared by many nesting sites. More genetic data would be desirable in the future, especially if improved genetic markers are used. These considerations and the coincidence of years with sudden increases of nests with years of expanse of the monitoring effort (e.g., in 2011 and 2016, when WWF efforts were increased and geographically extended) suggest that monitoring effort was at least a major cause of the observed results. Monitoring effort was low in mainland Sicily in the past, and often limited to nests discovered serendipitously [9]. As suggested by similar cases in the Mediterranean, an increase in monitoring can unveil important nesting sites [8,18].

Due to the length of coasts and limited personnel, the current monitoring effort cannot guarantee detection of all clutches laid. Given that most of nests have been reported by citizens, even in recent years with the highest monitoring effort, the nesting level in Sicily is likely still underestimated by current observations. We hypothesize that turtle nesting activity in Sicily is higher than other Italian sites such as Calabria and Pelagie Islands [8] and several times higher than currently observed.

There are 464 km of sandy shores suitable for turtle nesting along the coasts of Sicily. We identified three zones regarding nesting levels and sandy shores. The first zone is characterized by both long and short sandy shores with relatively high nesting levels (PUs E to L). Unlike what historical data suggested [8] present results show that the highest nesting activity occurs in the southeastern coast (PUs E to G) rather than in the central one (PUs H to L). The second zone is characterized by long continuous sandy shores with relatively low nesting levels reported (PUs T to D). The third zone is characterized by discontinuous sandy shores with relatively low nesting levels (PUs M to S). Here beaches are often small and fragmented, except for part of PU R, with several kilometers of sandy beach where nesting rarely occurred. In the latter two zones, nesting was mostly sporadic, both spatially and temporally. The first and the third zone most beaches have OC and YL sand, while in the second zone most beaches have WT and GR sand. Present results do not show a clear effect of sand color on nesting activity.

While nesting site availability can be reduced (e.g., by erosion), other environmental or anthropogenic factors discouraging nesting females are probably implicated where sandy beaches are available, such as strong currents of Messina Strait [19], pollution [20] and/or large harbors. However, it cannot be excluded that the observed lower nesting levels in this area represent an artifact due to a particularly low monitoring effort.

PUs F and G (Ragusa and Siracusa provinces) hosted the highest number of nests in the recent years. This was probably due to an intensive information campaign to tourists and local citizens about sea turtle tracks, their importance ad how to report them. In the future, the same activities should be carried out to other areas such as PUs N to R (Trapani and Palermo provinces) and PUs T to C (Messina Province), where the low number of nests observed so far may be due to a low research and information efforts. 

Coastal erosion represents a serious threat in several areas, such as PUs I to N, where many nests were lost due to erosion and inundation, and PUs T to A. In the latter area, coastal erosion is partly due to alteration of rivers due to coastal development and hence can be considered as an anthropogenic threat. Other beaches, especially in PUs F and G (Siracusa and Ragusa provinces) are reduced by the degradation of dunes due to constructions and sand mining.

Coastal development is a general and widespread threat, with a variety of negative effects to potential sea turtle reproductive habitats. In some areas, particularly in the PUs N to R, the very extent of sandy beaches has been reduced. In other areas (E to K), heavy cleaning vehicles are used daily during the summer season and can damage egg clutches, if present. Light pollution from artificial lights of roads, houses and touristic structures, known to cause disorientation of hatchlings [21,22], represent an important threat especially near large towns and in the eastern coast (PUs E to H).

Present results show that IP in Sicily (mean: 57 days) is generally higher than PIP and higher than most Mediterranean nesting sites (range of means: 45.6–59.6 days; [3]). A long IP may be due to low temperature outside the thermosensitive period for sex determination (approximately the middle third of development) [23] therefore IP is not an accurate indicator of sex ratio. However, the observed mean IP of the present sample size, that is longer than PIP reported by other studies, represents a preliminary indication of a male-biased ratio that needs to be confirmed through more accurate methods. The beach (or PU) appears to be a main factor affecting IP and probably sex ratio, with great variability of IP values among PUs. However, most of the investigated PUs can produce a wide range of IPs, and probably a wide range of sex ratios. Therefore, the entire island of Sicily probably has the potential to produce balanced or male-skewed sex ratios, differently from the typical female-skewed primary sex ratio in the Mediterranean and elsewhere [24], although confirmation through temperature and direct sex ratio data are needed. Several clutches were laid very early or late in the season, when IP > PIP is typical. These nests can produce more males [25] since they can be cooled by a general lower temperature compared to the peak of the nesting season [26], by rain or by more frequent cloudy days [27]. If a prevalence of males is produced in Sicily, this nesting area may become important for the Mediterranean population where a female-biased hatchlings production is suspected [28] and further feminization due to climate change is predicted [5]. In this respect, the two spatio-temporal factors affecting IP (PU and DATE) should be further investigated as potential factors affecting sex ratio.

Mean *HS* (63%) was generally lower compared to other important nesting sites such as Alagadi Beach, Cyprus (mean: 79.1; [29]), Zakynthos, Greece (range of means: 66.6–68.9; [30,31]) and compared to Calabria (mean: 86.0; [8]), although there is high variability between locations and, in the same location, between years. *HS* was affected by several factors of which PU and DIST are of potential interest for conservation since they can be targeted by specific management measures or conservation campaigns. Present results show a negative effect of relocation on *HS* and ES. The specific cause of such a difference could not be assessed. Relocation is a common practice aimed to increase *HS* in those clutches laid close to the water and at risk of inundation, that is known to decrease *HS* [32]. However, in comparison to natural clutches, both positive and negative effects of relocation on *HS* have been reported (reviewed by [33]). Relocation is a controversial practice when factors other than the simple *HS* are considered [34]. In particular, relocation may have negative consequences at population level since, even with lower *HS*, more males may be produced by inundated than non-inundated clutches [35]. Present results and the above considerations suggest relocating only clutches that would result in *HS* close to zero if left in place. In the perspective of a warming climate, where temperatures may rise to lethal levels for incubation [6,36], early or late nests such as those observed in Sicily could become of relevant conservation interest, as they could balance the low *HS* of the clutches incubating in warmer periods of the nesting season.

Regarding other reproductive parameters, temporal patterns of nesting and hatchling emergence largely overlap with those of other Mediterranean nesting sites in Turkey, Greece and Cyprus [18,29,31]. The observed mean clutch size was higher than in other Mediterranean sites with the exception of Greece where clutch size is even higher [3]. Such difference of clutch size among nesting sites may depend on different body size or different foraging areas [37]. Unfortunately, no information about these two parameters is available for females nesting in Sicily. Moreover, at present the foraging grounds of turtles nesting in Sicily are unknown. The positive correlation between CS and DIST represents an intriguing result of uncertain interpretation that requires further investigation.

Priority actions for conservation are: (i) identifying all nesting beaches suitable for turtle reproduction in Sicily through an expanded monitoring coverage, (ii) promoting the adoption by specific municipalities of protocols for minimizing anthropogenic impacts, such as correct management of beach resorts and reduction of light pollution, and (iii) informing the local communities and tourists about sea turtle conservation aspects, to support the previous two tasks. All of these tasks have been undertaken during the LIFE Euroturtles project (2017–2021), with an increased monitoring effort and the adoption of protocols by a few municipalities. These tasks should be continued, improved, and expanded geographically. Conservation measures can be facilitated when targeting the existing Natura 2000 sites, where 34.7% of all nesting events occurred, and additional Natura 2000 sites may be proposed in areas with high nesting activity.

## 5. Conclusions

Sicily is more important in loggerhead turtle nesting activity than previously thought and may even become more important in case of a future increase of nesting activity, especially in terms of producing balanced sex ratios in a climate change scenario. Given the extensive sandy coasts with scattered nesting, citizen science represents a fundamental complement to direct beach surveys in order to improve our knowledge on sea turtle nesting activity in Sicily. The present study identified PUs where monitoring effort and conservation measures should be promoted to guarantee suitable nesting habitats in the future. The primary sex ratio should be properly assessed in order to understand the importance of Sicily regarding production of males. The Sicily case shows the importance of investigating turtle nesting activity in areas with few or no records. Such an approach should be implemented in other Mediterranean coasts to improve the current limited picture of sea turtle nesting at Mediterranean level and its conservation implications.

## Figures and Tables

**Table 1:** Reproductive parameters of 217 natural and 23 relocated nests of loggerhead turtles found in Sicily from 1944 to 2021. The other 63 nests were reported for which these parameters were not available. IP, incubation period; CS, clutch size; *HS*, hatching success; ES, emergence success; DATE, week of nesting; IPMT, incubation period mean air temperature; DIST, distance of the nest from the shoreline; SC, sand color. GR, gray; OC, ochre; WH, white; YL, yellow.

Parameter	Natural		Relocated	
Mean min IP (*n*; Range; SD)	57 d (83; 36–80 d; 8.8 d)		58 d (19; 35–78 d; 10.1 d)	
Mean CS (*n*; Range; SD)	92.7 (155; 48–151; 20.4)		88.0 (23; 50–128; 20.1)	
Mean *HS* (*n*; Range; SD)	63% (155; 0–100%; 36.4%)		53% (23; 0–96.1%; 32.2%)	
Mean ES (*n*; Range; SD)	58.% (155; 0–100%; 35.6%)		47.9% (23; 0–95.5%; 32.6%)	
Mean DATE (*n*; Range; SD)	28.6 weeks (136; 22–33; 2.6) s		29 weeks (23; 26–34; 2.5)	
Mean IPMT (*n*; Range; SD)	28.2 °C (88;24.1–30.3 °C; 1.4 °C)		28.3 °C (20; 23.8–30.0 °C; 1.8 °C)	
Mean DIST (*n*; Range; SD)	20.3 m (140; 0–50 m; 10.2 m)		13.8 m (22; 2–30 m; 10 m)	
SC	GR	10	GR	1
	OC	130	OC	118
	WH	5	WH	1
	YL	72	YL	3

## Data Availability

The data presented in this study are available on request from the corresponding author.

## Supplementary Materials

The following supporting information can be downloaded at: https://www.mdpi.com/article/10.3390/ani12091221/s1. Figure S1: Distribution of nests with IP > PIP and IP < PIP; Figure S2: Scatterplot showing the relationship of CS and DIST; Figure S3: Predicted relationship between *HS* and independent variables. Table S1: Emergences; Table S2: Results of the fittest GLM for IP as dependent variable; Table S3: Results of the fittest GLM for *HS* as dependent variable. References [38,39,40,41,42,43,44,45,46,47,48,49,50,51,52,53,54,55,56,57] are cited in the supplementary materials.
