# Supplementary material for: Minor Sea Turtle Nesting Areas May Remain Unnoticed without Specific Monitoring: The Case of the Largest Mediterranean Island (Sicily, Italy)
Source: Animals (Basel). 2022 May 9;12(9):1221. doi: 10.3390/ani12091221 (PMC9101241; doi:10.3390/ani12091221)
Supplement: Supplementary file 1 [file animals-12-01221-s001.zip › animals-1695393-supplementary.pdf]

SUPPLEMENTAL MATERIAL

Minor sea turtle nesting areas may remain unnoticed without specific monitoring: the case of the largest Mediterranean island (Sicily, Italy)

Oleana Olga Prato[1], Valentina Paduano[1], Giulia Baldi[2], Salvatore Bonsignore[1], Gerlando Callea[1], Carlo Camera[1], Girolamo Culmone[3], Stefania D'angelo[1], Diego Fiorentino[1], Gino Galia[1], Salvatore Coriglione[1], Laura Genco[1], Giuseppe Mazzotta[1], Nicola Napolitano[1], Francesco Paolo Palazzo[1], Giuseppe Palilla[1], Dylan Pelletti[1], Toni Mingozzi[4], Luigi Agresti[1], Paolo Casale[2*]

[1] WWF Italy, Via Po, 25c, 00198, Roma, Italy
[2] Department of Biology, University of Pisa, via A. Volta 6, 56126, Pisa
[3] Associazione Caretta caretta, via L. Ariosto 86, 92031 Lampedusa, Italy
[4] DiBEST, Dept. of Biology, Ecology and Earth Sciences, Università della Calabria, P.te P. Bucci, Cubo 4/B, 87030 Rende CS, Italy

*Corresponding author: paolo.casale@unipi.it

Table S1. Emergences (Nests: N; False Crawls: FC) of loggerhead sea turtle in Sicily, from 1944 to 2021. For each event a reference is reported, for multiple references the number of events is in parentheses.

UF	Year	Locality #vp	N	FC	ITA	Type	Reference
G	1944	Scicli, Sampieri, RG	1		080008	ZSC	Fabrizi Daniele 1944 [7]
D	1960	Giarre, S. Anna, CT	1				Lino Luigi 1960 [14]
H	1963-1969	Scoglitti, Foce Ippari, RG	1				Mingozzi et al. 2007 [16]
I	1963-1969	Castarozzo di Palma, Marina di Palma, AG	2		040010	ZSC	Mingozzi et al. 2007 (3) [16]
I	1963-1969	Palma di Montechiaro, Marina di Palma, AG	1				Mingozzi et al. 2007 (3) [16]
F	1978-1983	Portopalo di Capopassero,SR	1		090003	ZSC	Mingozzi et al. 2007 [16]
H	1978-1983	Gela, CL	1				Mingozzi et al. 2007 [16]
N	1978-1983	Marsala, Petrosino, TP	1				Mingozzi et al. 2007 [16]
E	1985	Augusta, Agnone Bagni, SR	1				Mingozzi et al. 2007 [16]
F	1986	Noto, Vendicari, SR	1		090002<br>090027	ZSC<br>SIC	Mingozzi et al. 2007 [16]
H	1986	Gela, CL	1		050012	ZPS	Mingozzi et al. 2007 [16]
N	1986	Marsala, TP	1				Mingozzi et al. 2007 [16]
E	1987	Catania, Vaccarizzo, Villaggio nettuno, CT	1		070001<br>070029	ZSC<br>ZPS	Torrisi Andrea 1987 [18]
K	1990	Sciacca, Porto, TP	1				Mingozzi et al. 2007 [16]
M	1990	Mazara del vallo, Porto nuovo, TP	1				Mingozzi et al. 2007 [16]
I	1992	Palma di Montechiaro, Malerba, AG	1		040010	ZSC	Mingozzi et al. 2007 [16]
K	1993	Sciacca, Porto, TP	1				Mingozzi et al. 2007 [16]
L	1993	Menfi, Lido Fiori, AG	1		040012	SIC	Mingozzi et al. 2007 [16]
I	1994	Palma di Montechiaro, Marina di Palma, AG	1				Galia Franco 1994 [9]
K	1995	Siculiana,Torre salsa, AG	2		040003	ZSC	Mingozzi et al. 2007 (2) [16]

F	1996	Noto, Vendicari, SR	1		090002<br>090027	ZSC<br>SIC	Http://www.arcipescafisa.it/UserFiles/File/Newsletter_n30_Agosto10.pdf
H	1996	Scoglitti, Porto, RG	1				Mingozzi et al. 2007 [16]
J	1996	Realmonte, Punta Grande, AG	1				Mingozzi et al. 2007 [16]
M	1996	Mazara del vallo, Biscione, TP	1				Mingozzi et al. 2007 [16]
J	1999	Realmonte, Punta Grande, AG	1				Mingozzi et al. 2007 [16]
K	1999	Siculiana,Torre salsa, AG	1		040003	ZSC	Mingozzi et al. 2007 [16]
H	2000	Gela, CL	1				Mingozzi et al. 2007 [16]
G	2002	Punta delle Formiche	1		090003	ZSC	Local citizen 2002 [15]
J	2002	Porto empedocle, Villa Romana, AG	1				Mingozzi et al. 2007 [16]
K	2003	Sciacca, Porto, TP	1				Mingozzi et al. 2007 [16]
K	2005	Realmonte, Giallonardo, AG	2				Galia et al. 2006 (2) [10]
L	2006	Menfi, Mandrarossa, AG	1				http://www.wwftorresalsa.it/02_08_06-html
E	2007	Augusta, Agnone, SR	1				Torrisi Andrea 2007 [18]
N	2007	Custonaci, Cornino, TP	1		010025	SIC	Genco et al. 2008 [11]
K	2008	Realmonte, Giallonardo, AG	1				http://www.adnkronos.com/IGN/Cronaca/?id=3.0.2589567347
K	2008	Siculiana, Siculiana marina, AG	1				Present study
F	2010	Avola, Piccio, SR	1				Insacco et al. 2011 [12]
F	2010	Noto, Lido di noto, SR	2				Insacco et al. 2011 (2) [12]
F	2010	Noto, Vendicari, SR	1	1	090002<br>090027	ZSC<br>SIC	https://www.scomunicando.it/notizie/tartaruga-caretta-caretta-depone-uova-a-vendicari-e-linosa-2/; Http://www.arcipescafisa.it/UserFiles/File/Newsletter_n30_Agosto10.pdf
F	2010	Noto, S. Lorenzo, SR	1				https://sikulamente.wordpress.com/tag/tartarughe/
S	2010	Pollina, Finale di Pollina, PA	1				Insacco et al. 2011 [12]
G	2011	Ragusa, Torre Cabrera, RG	1				http://www.ragusaoggi.it/11498/
J	2011	Realmonte, Punta Grande, AG	1				Casale et al. 2012 [3]
K	2011	Realmonte, Giallonardo, AG	4				Casale et al. 2012 (4) [3]
L	2011	Sciacca, Capo San Marco, AG	1		040012	SIC	http://sciacca.agrigentonotizie.it/cronaca/sciacca-si-schiudono-uova-di-caretta-car_62
L	2011	Menfi, Porto Palo, AG	1				Casale et al. 2012 [3]
Q	2011	Palermo, Acqua dei corsari, PA	1				Casale et al. 2012 [3]
E	2012	Catania, Plaja di Catania, CT	1				Http://www.pro-natura.it/files/redazione/natura-e-societa/2012-03.pdf

E	2012	Augusta, Agnone Bagni, SR	1				Http://www.pro-natura.it/files/redazione/natura-e-societa/2012-03.pdf
K	2012	Realmonte, Giallonardo, AG	1				http://www.wwf.it/client/stampa.aspx
K	2012	Eraclea minoa, CapoBianco, AG	1		040003	ZSC	http://www.comunicalo.it/index.php?option=com_content&view=article&id=11832
M	2012	Mazara del vallo, Tonnarella, TP	1				http://www.mazaraonline.it/?p=41236&print=1
A	2013	Messina, Venetico, ME	1				Isgrò Carmelo 2013 [13]
F	2013	Avola, Cicerata, SR	2				Zaffarana Rosario 2013 (1) [19]<br>Belfiore Vincenzo 2013 (1) [1]
F	2013	Noto, Vendicari, SR	1		090002<br>090027	ZSC<br>SIC	http://www.siracusanews.it/node/39561, http://www.ilsitodipalermo.it/content/522-sessantatre-uova-di-caretta-caretta-si-schiudono-nelloasi-di-vendicari
K	2013	Siculiana, Siculiana marina, AG	1				http://www.agrigentonotizie.it/speciale/earth-day/Siculiana-Marina-trovato-primo-nido-tartarughe-marine.html
L	2013	Sciacca, Capo San Marco, AG	3		040012	SIC	http://www.wwf.it/news/notizie/?2901 (2);http://www.agrigentoflash.it/2011/09/02/sciacca-la-carica-delle-80-caretta-caretta/ (1)
Q	2013	Palermo, Mondello Lido Ombelico del mondo, PA	1				Present study; (http://www.siciliafan.it/tartaruga-depone-60-uova-a-mondello/
E	2014	Augusta, Agnone, SR	1				Http://www.pro-natura.it/files/redazione/natura-e-societa/2014-4.pdf
E	2014	Catania, Plaja di Catania, CT	1				https://tanogabo.com/alcuni-nidi-di-tartarughe-di-mare-in-sicilia/
F	2014	Noto, S. Lorenzo, SR	1				Http://www.pro-natura.it/files/redazione/natura-e-societa/2014-4.pdf
G	2014	Ispica, Santa Maria del Focallo, RG	1				http://www.corrierediragusa.it/articoli/attualit%E0/ispica/27970-caretta-caretta-appena-nate-falcidiate-da-trattore-comunale.html
L	2014	Menfi, Capparrina, AG	1				http://www.wwf.it/news/?9140/Menfi-volontari-WWF-scoprono-nido-di-tartaruga-marina
L	2014	Campobello di mazara, Tre Fontane, TP	2		010011	ZSC	http://www.marsalaviva.it/cronaca/cronaca-della-provincia/item/16481-nate-59-tartarughe-nella-spiaggia-di-tre-fontane; http://www.castelvetranoselinunte.it/oltre-40-tartarughe-nate-a-tre-fontane-per-triscina-si-attende/84779/
M	2014	Mazara del vallo, Tonnarella, TP	1				http://www.wwf.it/news/notizie/?9860, http://qn.quotidiano.net/lifestyle/2014/07/31/1086607-animali-tartaruga-wwf.shtml, http://qn.quotidiano.net/lifestyle/2014/07/04/1085375-animali-nido-caretta.shtml
E	2015	Catania, Plaja di Catania, CT	2				Present study

E	2015	Oasi del Simeto, Villaggio Campo di Mare, CT	1				Present Study
F	2015	Portopalo di Capopassero, Isola delle Correnti, SR	1		090003	ZSC	Scala Santino 2015 [17]
J	2015	Agrigento, Zingarello, AG	1				http://guidominciotti.blog.ilsole24ore.com/2015/08/22/sorpresa-a-zingarello-inattesa-posa-e-schiusa-di-uova-di-tartarughe-caretta-caretta/?refresh_ce=1
K	2015	Siculiana,Torre salsa, AG	2		040003	ZSC	http://www.wwf.it/petrolio_mi_sta_stretto.cfm?17320/Nuovo-nido-di-tartaruga-a-Torre-Salsa; https://www.siculiana.info/2015/07/nuovo-nido-di-caretta-caretta-nella.html
L	2015	Sciacca, Capo San Marco, AG	3		040012	SIC	http://www.vita.it/it/article/2015/07/22/nuovi-nidi-di-tartaruga-marina-a-sciacca/136031/ ; https://www.meteoweb.eu/2015/07/le-tartarughe-caretta-caretta-preferiscono-sciacca-2-nidi-scoperti-dal-wwf/473503/; http://www.agrigentonotizie.it/cronaca/secondo-nido-caretta-caretta-localita-san-marco-sciacca-4-agosto-2015.html
E	2016	Oasi del Simeto, San Francesco La Rena, CT	1				Present study
E	2016	Oasi del Simeto, Villaggio Gabbiano BLU,CT	2	1	070001<br>070029	ZSC<br>ZPS	Present study
E	2016	Catania, Vaccarizzo,CT		1	070001<br>070029	ZSC<br>ZPS	Present study
F	2016	Siracusa, Arenella, SR	2				Cavarra Daniela 2016 (2) [4]
F	2016	Siracusa, Fontane Bianche, SR	1				http://www.siracusanews.it/node/74516
F	2016	Avola, SR	3				Https://www.entefaunasiciliana.it/wp-content/uploads/delightful-downloads/2020/04/Grifone-134-ANNO-XXV-n.-4-31-agosto-2016.pdf (3)
F	2016	Noto, Lido di Noto, SR	2				Falesi Laura 2016 (1) [8]<br>Https://www.entefaunasiciliana.it/wp-content/uploads/delightful-downloads/2020/04/Grifone-134-ANNO-XXV-n.-4-31-agosto-2016.pdf (1)
F	2016	Noto, Vendicari, SR	1	3	090002<br>090027	ZSC<br>SIC	Https://www.entefaunasiciliana.it/wp-content/uploads/delightful-downloads/2020/04/Grifone-134-ANNO-XXV-n.-4-31-agosto-2016.pdf (3 FC);<br>https://www.entefaunasiciliana.it/wp-content/uploads/delightful-downloads/2020/04/Grifone-135-ANNO-XXV-n.-5-6-24-dicembre-2016.pdf (1 N)
F	2016	Noto, S. Lorenzo, SR	2				Present study
F	2016	Pachino, Morghella, SR	1				Https://www.entefaunasiciliana.it/wp-content/uploads/delightful-downloads/2020/04/Grifone-134-ANNO-XXV-n.-4-31-agosto-2016.pdf

G	2016	Pachino, Costa dell'ambra, SR	1		090003	ZSC	Betulla Alessandra 2016 [2]
G	2016	Ispica, Santa Maria del Focallo, RG	1				http://ragusa.gds.it/2016/07/31/una-caretta-caretta-sceglie-ispica-e-depone-le-sue-uova-al-focallo_546526/
G	2016	Pozzallo, Pietrenere, RG	2				http://www.lasicilia.it/news/home/23295/sulla-spiaggia-di-avola-uova-di-caretta-caretta-e-due-delfini-morti.html; http://meridionews.it/articolo/45133/pozzallo-caretta-caretta-deposita-le-uova-a-pietrenere-eravamo-in-spiaggia-labbiamo-vista-uscire-dallacqua/
G	2016	Santa croce camerina, Punta Braccetto, RG	1				http://www.nuovosud.it/41276-ambiente-ragusa/punta-braccetto-tartaruga-depone-le-uova-sulla-spiaggia-di-montalbano
J	2016	Porto empedocle, AG	1				http://www.agrigentonotizie.it/cronaca/porto-empedocle-salvata-piccola-caretta-caretta.html
L	2016	Sciacca, Capo San Marco, AG		1	040012	SIC	http://agrigento.gds.it/2016/10/08/nidi-di-tartarughe-a-sciacca-e-menfi-cresce-lattesa-per-la-schiusa_574328/
L	2016	Menfi, Porto Palo, AG	1				http://www.agrigentonotizie.it/cronaca/menfi-nascono-tartartughe-caretta-caretta-porto-palo.html
L	2016	Menfi, Lido Fiori, AG	1		040012	SIC	http://www.agrigentonotizie.it/cronaca/tartaruga-caretta-caretta-nidifica-menfi.html
L	2016	Triscina, Selinunte, TP	1		010011	ZSC	http://www.castelvetranoselinunte.it/triscina-breve-la-schiusa-delle-tartarughe-presidi-notte/85104/
L	2016	Campobello di mazara, Tre Fontane, TP	1		010011	ZSC	http://www.castelvetranoselinunte.it/triscina-breve-la-schiusa-delle-tartarughe-presidi-notte/85104/
R	2016	Lascari, PA	1				https://www.wwfsicilianordoccidentale.it/2016/07/28/salvaguardia-delle-tartarughe-marine/
T	2016	Naso, Ponte Naso, ME	1				Present study
U	2016	Patti, C. da Galige, ME		1			Present study
E	2017	Oasi del Simeto, Villaggio Primosolo II; CT	1	1	070001<br>070029	ZSC<br>ZPS	Present study
E	2017	Vaccarizzo,CT	1		070001<br>070029	ZSC<br>ZPS	Present study
E	2017	Augusta,Agnone Bagni, Villaggio Porto San Leonardo, SR	1				Present study
F	2017	Noto, Lido di Noto, SR	1	1			Present study
F	2017	Noto, Vendicari, SR		5	090002<br>090027	ZSC<br>SIC	Present study

F	2017	Noto, S. Lorenzo, SR		2			Present study
F	2017	Portopalo di Capopassero, Isola delle Correnti, SR	1		090003	ZSC	Present study
G	2017	Ispica, Santa Maria del Focallo, RG	3				Di Rosa Antonio 2017 (3) [6]
G	2017	Pozzallo, RG	2				Present study
G	2017	Modica, Marina di modica, RG	1		080008	ZSC	Present study
G	2017	Scicli, Cava d'Aliga, RG		1			Present study
H	2017	Scoglitti, Sabbie d'oro, RG	1				Present study
I	2017	Licata, Marianello, AG	1				Present study
K	2017	Siculiana,Torre salsa, AG	2	1	040003	ZSC	Present study
K	2017	Sciacca, Renella, AG		1	040012	SIC	Present study
L	2017	Sciacca, Capo San Marco, AG	1		040012	SIC	Present study
L	2017	Menfi, Capparrina, AG	2		040012	SIC	Present study
S	2017	Capo d'Orlando, ME		1			Present study
D	2018	Calatabiano, CT	1	1			Present study
E	2018	Catania, Oasi del Simeto, CT		1	070001<br>070029	ZSC<br>ZPS	Present study
E	2018	Catania, Vaccarizzo,CT	1		070001<br>070029	ZSC<br>ZPS	Present study
F	2018	Priolo, Marina di Melilli, SR	1				Present study
F	2018	Avola, SR	2	3			Present study
F	2018	Noto, Vendicari, SR	1	6	090002<br>090027	ZSC<br>SIC	Present study
F	2018	Noto, S. Lorenzo, SR	1				Present study
F	2018	Portopalo di Capopassero, Isola delle Correnti, SR	2		090003	ZSC	Present study
G	2018	Pachino, Costa dell'ambra, SR	1		090003	ZSC	Present study
G	2018	Ispica, Santa Maria del Focallo, RG	5				Present study
G	2018	Scicli, Cava d'Aliga, RG		1			Present study
I	2018	Palma di montechiaro, Contrada Ciotta, AG	1				Present study
I	2018	Licata, Cala del Re, AG	1				Present study

K	2018	Siciliana, Siculiana marina, La scogliera, AG	1				Present study
K	2018	Torre salsa, Loc. Fungicedda, AG		1	040003	ZSC	Present study
L	2018	Triscina, TP	1		no	Map	Present study
N	2018	Marsala, Tp	2				Present study
N	2018	Trapani, Spiaggia della Tipa, TP	1		010007	ZSC	Present study
N	2018	Erice, San Giuliano, TP	2				Present study
N	2018	Valderice, TP		1	010025	SIC	Present study
R	2018	Lascari, Salinelle, PA		1			Present study
D	2019	ACIREALE,SANTA TECLA, CT		1			Present study
E	2019	Catania, Plaja di Catania, CT	1				Present study
F	2019	Avola, Marina di Avola, SR		1			Present study
F	2019	Noto, Lido di Noto, SR	2				Present study
F	2019	Noto, Vendicari, SR		3	090002<br>090027	ZSC<br>SIC	Present study
F	2019	Noto, S. Lorenzo, SR	1	1			Present study
F	2019	Pachino, Morghella, SR	4				Present study
F	2019	Portopalo di Capopassero, Isola delle Correnti, SR	5	2	090003	ZSC	Present study
G	2019	Ispica, Santa Maria del Focallo, RG	3	1			Present study
G	2019	Modica, Marina di modica, RG	1		080008	ZSC	Present study
G	2019	Scicli, Sampieri, RG	2		080008	ZSC	Present study
G	2019	Scicli, Donnalucata, RG	1	1			Present study
H	2019	Scoglitti, Foce Ippari, RG	1				Present study
J	2019	Agrigento, Cannatello, AG	1				Present study
J	2019	Realmonte, Punta Grande, AG		1			Present study
K	2019	Siculiana, Siculiana marina, AG	4	1			Present study
K	2019	Siculiana,Torre salsa, AG	1		040003	ZSC	Present study
L	2019	Sciacca, Capo San Marco, AG	2		040012	SIC	Present study
L	2019	Menfi, Porto Palo, AG	1				Present study
L	2019	Menfi, lido fiori, AG		1	040012	SIC	Present study

L	2019	Triscina, TP	1		010011	ZSC	Present study
R	2019	Cefalù, PA	1				Present study
U	2019	Milazzo, ME	1				Isgrò Carmelo 2019 [13]
A	2020	Messina, Santo Saba, ME	1				Present study
B	2020	Giampilieri, Giampilieri marina, ME		1			Present study
D	2020	Calatabiano, CT	1				Present study
D	2020	Acireale, CT		3			Present study
E	2020	Catania, Plaja di Catania, CT	1				Present study
E	2020	Catania, Oasi del Simeto, CT	1		070001<br>070029	ZSC<br>ZPS	Present study
E	2020	Catania, Vaccarizzo,CT	1	1	070001<br>070029	ZSC<br>ZPS	Present study
E	2020	Augusta, Agnone, SR	2	1			Present study
F	2020	Priolo, Marina di Melilli, SR	1				Cilea Fabio 2020 [5]
F	2020	Siracusa, Arenella, SR	1				Present study
F	2020	Siracusa, Ognina, SR	3				Present study
F	2020	Siracusa, Fontane Bianche, SR		1			Present study
F	2020	Avola, SR	7	12			Present study
F	2020	Noto, Lido di Noto, SR	5	5			Present study
F	2020	Noto, Vendicari, SR	1	1	090002<br>090027	ZSC<br>SIC	Present study
F	2020	Noto, S. Lorenzo, SR	7	1			Present study
F	2020	Pachino, La Plage Santomar, SR	1		090004	ZSC	Present study
F	2020	Portopalo di Capopassero, Scalo Mandrie, SR	2	1	090028	SIC	Present study
F	2020	Portopalo di Capopassero, Isola delle Correnti, SR	11	11	090003	ZSC	Present study
G	2020	Pachino, Punta delle Formiche, SR		1	090003	ZSC	Present study
G	2020	Pachino, Granelli, SR	1				Present study
G	2020	Ispica, Santa Maria del Focallo, RG		1			Present study
G	2020	Modica, Marina di modica, RG	6	6	080008	ZSC	Present study
G	2020	Scicli, Sampieri, RG	1	1	080008	ZSC	Present study
G	2020	Scicli, Cava d'Aliga, RG	1	1			Present study

G	2020	Scicli, Donnalucata, RG	1	1			Present study
G	2020	Scicli, Plaia Grande, RG	1		080001<br>080010	ZSC<br>SIC	Present study
G	2020	Ragusa, Marina di Ragusa, RG		1			Present study
G	2020	Santa croce camerina, Casuzze,RG	2	2			Present study
G	2020	Santa croce camerina, Punta Secca, RG		1			Present study
H	2020	Santa croce camerina, Randello, RG	3	5	080004	ZSC	Present study
H	2020	Scoglitti, Baia Dorica, RG	1				Present study
H	2020	Scoglitti, Kamarina, RG		1			Present study
I	2020	Licata, Pisciotto, AG	1				Present study
I	2020	Licata, Foce Gallina, AG		4			Present study
I	2020	Licata, Marianello, AG		1			Present study
J	2020	Agrigento, Cannatello, AG	1				Present study
J	2020	San Leone, Le Dune, AG	1				Present study
J	2020	Porto empedocle, AG	3				Present study
J	2020	Realmonte, Punta Grande, AG	1	1			Present study
K	2020	Realmonte, Giallonardo, AG	2				Present study
K	2020	Siculiana, Siculiana marina, AG	3				Present study
K	2020	Siculiana,Torre salsa, AG	2		040003	ZSC	Present study
K	2020	Ribera, Borgo bonsignore, AG	1		040003	ZSC	Present study
O	2020	San vito lo capo, TP		3			Present study
O	2020	Alcamo Marina-Battigia, TP	1				Present study
O	2020	Alcamo Marina-Calatubo, TP	1		010018	ZSC	Present study
O	2020	Trappeto, PA	1				Present study
S	2020	Capo d'Orlando, ME	1				Present study
T	2020	Naso, Ponte Naso, ME		1			Present study
T	2020	Brolo, ME	1				Present study
T	2020	Gliaca di Piraino, ME	2	2			Present study
T	2020	Gioiosa Marea, Capo Calavà, ME		7	030033	ZSC	Present study

U	2020	Patti, Marina di Patti, ME		1			Present study
U	2020	Milazzo, ME		1			Isgrò Carmelo 2020 [13]
A	2021	Villafranca Tirrena, Saponara marittima, ME		2			Present study
C	2021	Roccalumera, Hotel Main, ME		1			Present study
D	2021	Calatabiano, CT		1			Present study
E	2021	Catania, Plaja di Catania, CT	1				Present study
E	2021	Augusta, Agnone Bagni, SR	1	2			Present study
F	2021	Augusta, Paradiso, SR	1	1			Present study
F	2021	Siracusa, Arenella, SR	1				Present study
F	2021	Avola, SR	9	3			Present study
F	2021	Noto, Lido di Noto, SR	5	6			Present study
F	2021	Noto, Vendicari, SR	4	1	090002<br>090027	ZSC<br>SIC	Present study (2 N e 1 FC) Ente Fauna Siciliana asscociation (2 N)
F	2021	Pachino, Morghella, SR	1	1			Present study
F	2021	Portopalo di Capopassero, Isola delle Correnti, SR	6	8	090003	ZSC	Present study
G	2021	Pachino, Granelli, SR	3	19			Present study
G	2021	Pachino, Punta delle Formiche, SR	1	1	090003	ZSC	Present study
G	2021	Modica, Marina di modica, RG	1		080008	ZSC	Present study
G	2021	Scicli, Sampieri, RG	1		080008	ZSC	Present study
G	2021	Scicli, Cava d'Aliga, RG	1				Present study
G	2021	Ragusa, Marina di Ragusa, RG	2				Present study
H	2021	Santa croce camerina, Casuzze,RG	1				Present study
H	2021	Butera, Marina di Butera, CL	1		050012	ZPS	Present study
I	2021	Licata, Pisciotto, AG	1				Present study
J	2021	Porto empedocle, AG	3				Present study
K	2021	Siculiana, Siculiana marina, AG	1				Present study
L	2021	Sciacca, Capo San Marco, AG	1		040012	SIC	Present study
N	2021	Marsala, Petrosino, TP	2	1			Present study
N	2021	Castelluzzo, TP	1		010029	ZPS	Present study
O	2021	Balestrate, PA		1			Present study

O	2021	Trappeto, Ciamarrita, PA	1				Present study
R	2021	Casteldaccia, PA		1			Present study
T	2021	Gioiosa Marea,Capo Calavà, ME		1	030033	ZSC	Present study
U	2021	Milazzo, ME		1			Present study
U	2021	Patti, Laghetti marianello, ME		2	030012	ZSC	Present study

REFERENCES

1. Belfiore V, Legambiente val di noto association, Noto, Siracusa, Italy. Personal communications. (2013)
2. Betulla A, Beach Owner, Pachino, Siracusa, Italy. Personal Communications. (2016)
3. Casale, P, Palilla, G, Salemi, A, Napoli, A, Prinzi, M, Genco, L, et al. (2012) Exceptional sea turtle nest records in 2011 suggest an underestimated nesting potential in Sicily (Italy). Acta Herpetologica, 7(1):181-188.
4. Cavarra D, Beach owner, Siracusa, Italy. Personal communicatons. (2016)
5. Cilea F, Lipu association Saline di Priolo, Priolo Gargallo, Siracusa, Italy. Personal communications. (2020)
6. Di Rosa A, Wwf Italy, Modica, Ragusa, Italy. Personal Communications. (2017)
7. Fabrizi D, Local citizen, Sampieri, Scicli, Ragusa, Italy. Personal communications. (1944)
8. Falesi L, Archeo club association Val di noto, Noto, Siracusa, Italy. Personal communications. (2016)
9. Galia F, WWF Italy, Licata, Agrigento, Italy. Personal communications. (1994)
10. Galia, F, Freggi, D, d'Angelo, S, Lo Valvo, M. (2006) An unusual nest activity along southern Sicilian coasts: an hope for sea turtle survival? In: 26th Annual Symposi-um on Sea Turtle Biology and Conservation, 295-296.
11. Genco, L, Culmone, G, Cortellaro, G, Federico, R, Piacentino, A, Prinzi, M. (2008) Prima segnalazione di nidificazione di tartaruga comune *Caretta caretta* in Sicilia nord-occidentale (Italia meridionale). Atti Soc. it. Sci. nat. Mus. civ. Stor. nat. Milano 149:325-330.
12. Insacco G, Inclimona V, Barlotta A, Ricotta V, Denise G, Spadola F, Blasi MF, Scaravelli D. (2011) The activity of the Sea Turtle rehabilitation in the Regional Rescue Center of Comiso (Sicily, Ragusa, Italy) in 10 years. 4th Mediterranean Conference on Marine Turtles – Napoli.
13. Isgrò C, MuMa museum, Milazzo, Messina, Italy. Personal communications. (2013) (2019) (2020)
14. Lino L, Pronatura association, Catania, Italy. Personal communications. (1960)
15. Local Citizen, Pachino, Siracusa, Italy. Personal Communications (2002)
16. Mingozzi, T, Masciari, G, Paolillo, G, Pisani, B, Russo, M, Massolo, A. (2007) Discovery of a regular nesting area of loggerhead turtle *Caretta caretta* in southern Italy: a new perspective for national conservation. Biodivers. Conserv. 16: 3519-3541.
17. Scala S, Beach owner, Portopalo di Capopassero, Siracusa, Italy. Personal Communications. (2015)
18. Torrisi A, Local citizen, Catania,Italy. Personal communications. (1987) (2007)
19. Zaffarana R, Local citizen, Avola, Siracusa, Italy. Personal communications. (2013)

Table S2. Results of the fittest GLM for IP as dependent variable. Only variables (or levels) with p<0.05 are shown. PU F, YEAR 2020, SC OC are the first (intercept) levels (n = 74).

	Estimate	Std. Error	T value	P
(Intercept)	1.07E-01	4.48E-02	2.382	0.02038
PUG	-1.43E-03	6.12E-04	-2.341	0.02255
DATE	-4.53E-03	1.60E-03	-2.83	0.00632
IPMT	-3.49E-03	1.62E-03	-2.149	0.03568
DATE:IPMT	1.73E-04	5.79E-05	2.99	0.00404

Table S3. Results of the fittest GLM for HS as dependent variable. Only variables (or levels) with p<0.05 are shown. PU F, YEAR 2020, SC OC are the first (intercept) levels (n = 74).

	Estimate	Std. Error	z value	P
(Intercept)	11.42435	4.169888	2.74	0.006149
PUG	-0.17182	0.067368	-2.55	0.010759
PUH	0.696577	0.154689	4.503	6.70E-06
PUI	0.803656	0.153202	5.246	1.56E-07
PUN	0.374499	0.147379	2.541	0.011052
PUO	-0.33363	0.093424	-3.571	0.000356
YEAR2017	0.240395	0.084853	2.833	0.00461
YEAR2018	0.550058	0.067571	8.14	3.94E-16
YEAR2019	0.4774	0.061948	7.706	1.29E-14
DATE	-0.41289	0.145305	-2.842	0.004489
IPMT	-0.41312	0.15034	-2.748	0.005998
SCYL	0.293127	0.050598	5.793	6.90E-09
DIST	0.005254	0.0024	2.19	0.028547
CS	-0.00839	0.001047	-8.016	1.09E-15
DATE:IPMT	0.016023	0.005214	3.073	0.002117

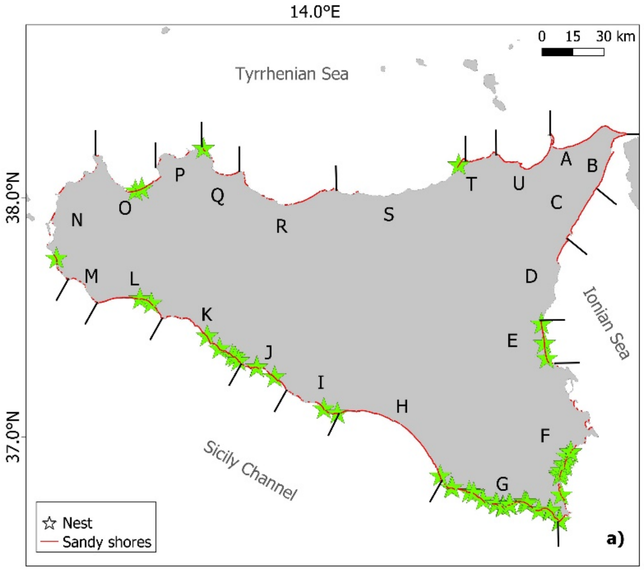

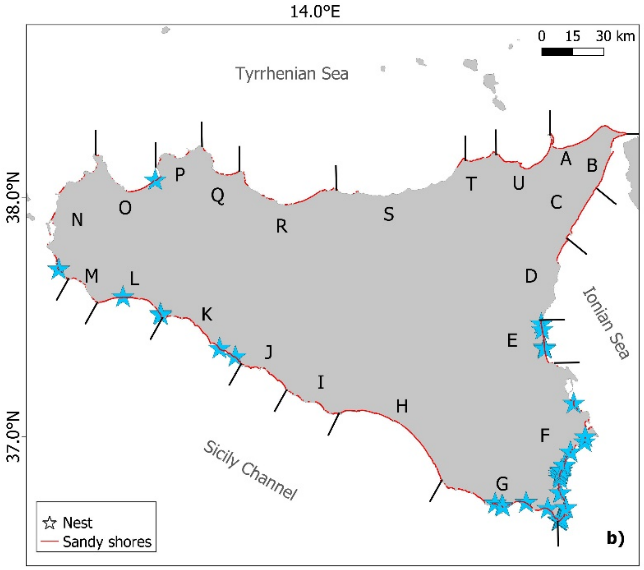

Figure S1. Distribution of nests with IP>PIP, potentially producing more males (a, n = 55), and IP<PIP, potentially producing more females (b, n = 50).

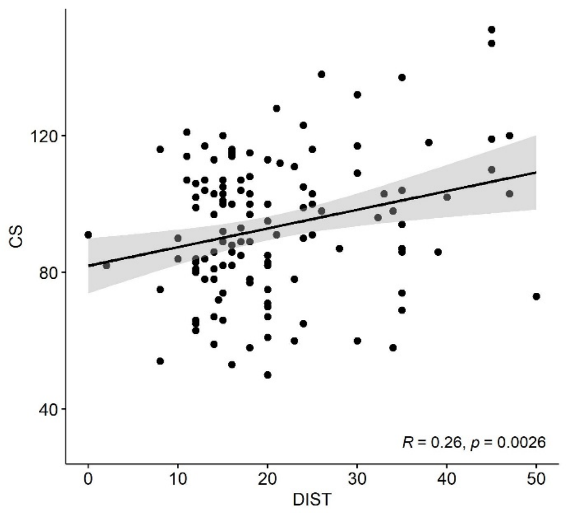

Figure S2. Scatterplot showing the relationship of CS and DIST, black line represents the regression line with 95% CI in light gray. Regression coefficient and P-value resulting from the Pearson chi-squared test are shown. (n = 127)

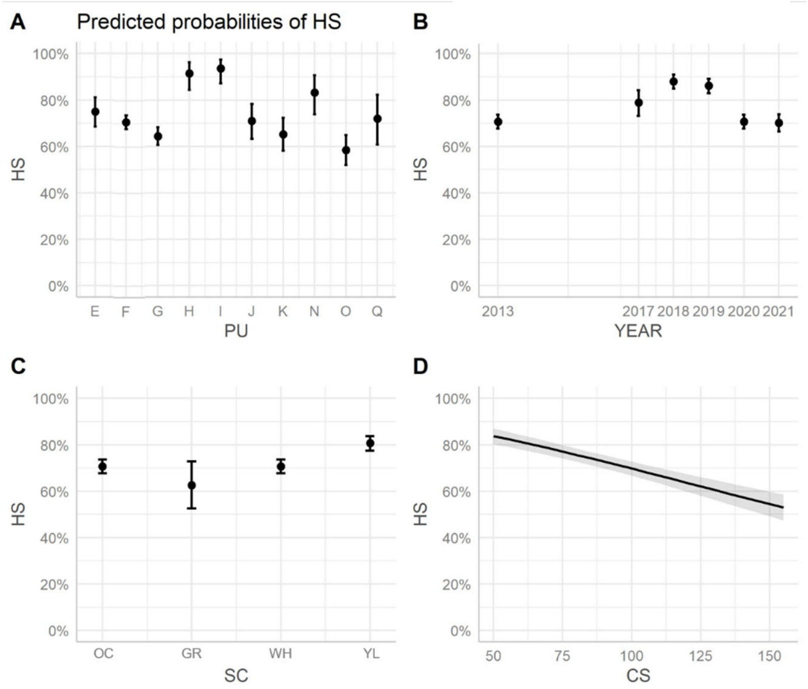

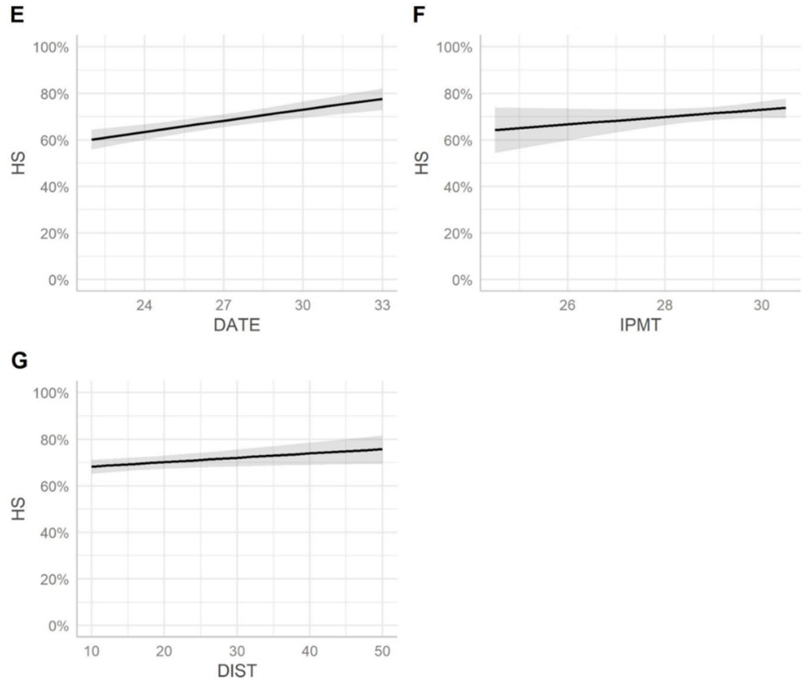

Figure S3. Predicted values of HS (estimated marginal means) of loggerhead turtle nests in Sicily plotted separately to show the effect of each IV, originated from the fittest GLM model. Dots in plot A-C show predicted value for each PU, YEAR, SC, whiskers represent 95% CI. Plot D-G represent regression lines based on the GLM model with 95% CI in light gray. SC: sand colour (OC: Ochre, GR: Gray, WH: White, YL: Yellow). CS: clutch size (number of eggs). DATE: date of nesting (week of the year). IPMT: mean air temperature during the incupation period (°C). DIST: distance of the nest from the shoreline (m).
